# The Isolation and Characterization of β-Glucogallin as a Novel Aldose Reductase Inhibitor from *Emblica officinalis*

**DOI:** 10.1371/journal.pone.0031399

**Published:** 2012-04-02

**Authors:** Muthenna Puppala, Jessica Ponder, Palla Suryanarayana, Geereddy Bhanuprakash Reddy, J. Mark Petrash, Daniel V. LaBarbera

**Affiliations:** 1 Department of Biochemistry, National Institute of Nutrition, Hyderabad, India; 2 Department of Pharmaceutical Sciences, Skaggs School of Pharmacy and Pharmaceutical Sciences, University of Colorado Anschutz Medical Campus, Aurora, Colorado, United States of America; 3 Department of Ophthalmology, School of Medicine, University of Colorado Anschutz Medical Campus, Aurora, Colorado, United States of America; Medical College of Wisconsin, United States of America

## Abstract

Diabetes mellitus is recognized as a leading cause of new cases of blindness. The prevalence of diabetic eye disease is expected to continue to increase worldwide as a result of the dramatic increase in the number of people with diabetes. At present, there is no medical treatment to delay or prevent the onset and progression of cataract or retinopathy, the most common causes of vision loss in diabetics. The plant *Emblica officinalis* (gooseberry) has been used for thousands of years as a traditional Indian Ayurvedic preparation for the treatment of diabetes in humans. Extracts from this plant have been shown to be efficacious against the progression of cataract in a diabetic rat model. Aldose reductase (ALR2) is implicated in the development of secondary complications of diabetes including cataract and, therefore, has been a major drug target for the development of therapies to treat diabetic disease. Herein, we present the bioassay-guided isolation and structure elucidation of 1-*O*-galloyl-β-D-glucose (β-glucogallin), a major component from the fruit of the gooseberry that displays selective as well as relatively potent inhibition (IC_50_ = 17 µM) of AKR1B1 *in vitro*. Molecular modeling demonstrates that this inhibitor is able to favorably bind in the active site. Further, we show that β-glucogallin effectively inhibits sorbitol accumulation by 73% at 30 µM under hyperglycemic conditions in an *ex-vivo* organ culture model of lenses excised from transgenic mice overexpressing human ALR2 in the lens. This study supports the continued development of natural products such as β-glucogallin as therapeutic leads in the development of novel therapies to treat diabetic complications such as cataract.

## Introduction

Diabetes mellitus is recognized as a leading cause of new cases of blindness throughout the world, and the rapid increase in the incidence of diabetes in recent years suggests that diabetic eye disease could become an even larger public health problem in the near future [1]. Diabetic patients face a 25-fold increased risk of blindness as a result of diabetic retinopathy and/or cataract in comparison with the general population. While strict long term control of blood glucose can reduce the likelihood of developing retinal lesions leading to retinopathy [2], present methods for achieving strict metabolic control are not suitable for most diabetic patients because of excessive cost and complexity. Therefore, patient education, lifestyle modifications, and new technologies such as blood glucose monitors and insulin pumps collectively will still fall short of effectively preventing diabetic eye disease for the general population. Numerous clinical trials and experimental animal studies have shown that early intervention is required to achieve maximal reduction in the onset and severity of diabetic retinopathy and cataracts [2], [3]. Therefore, medical therapies developed to delay the onset and progression of diabetic eye disease must be sufficiently safe and well tolerated to allow lifelong treatment.

Many theories have been advanced to explain the pathogenesis of diabetic eye disease. These include excess formation of advanced glycation end-products (AGEs), activation of the glucosamine pathway, activation of PKC isoforms, and activation of the polyol pathway [4]. The first step of the polyol pathway is catalyzed by aldose reductase, which converts glucose to sorbitol with concomitant oxidation of NADPH to NADP^+^ (Note: ALR2 will be used in generic reference to aldose reductase. In cases referring to aldose reductase of a defined species origin, we will use the standard nomenclature adopted for the aldo-keto reductase superfamily, such as AKR1B1 for human aldose reductase. ALR1 will be used in generic reference to aldehyde reductases). Accelerated flux of glucose through the polyol pathway has been implicated in the pathogenesis of diabetic eye disease. Several groups have reported that ALR2 becomes activated in diabetic tissues [5]–[7]. We recently showed that elevated ALR2 activity measured in erythrocytes was associated with risk for developing retinopathy among patients with type 2 diabetes [8]. Enhancement of ALR2 activity by creating transgenic animals causes exacerbation of diabetic eye disease, including cataract [9] and retinopathy [10], [11]. In contrast, inactivation of the ALR2 gene by targeted gene deletion protects against diabetes-induced cataract and histopathological markers of retinopathy such as pericyte loss, blood-retinal barrier breakdown, increased VEGF, and markers of retinal nitrosative stress [12].

Given the close association between ALR2-mediated sorbitol accumulation and diabetic eye disease, considerable effort has been focused on developing ALR2 inhibitors to prevent diabetic retinopathy. Although several structurally diverse inhibitors have been studied clinically, none have been shown to prevent the onset or worsening of diabetic retinopathy in humans. In contrast, impressive results have been reported with several different ALR2 inhibitors against markers of diabetic retinopathy in animal models. ALR2 inhibitors essentially prevent cataract [11], retinal pericyte loss and the formation of acellular capillaries in diabetic animal models [13], [14]. These results appear to validate ALR2 as an attractive target against diabetic eye disease and suggest that development of more effective inhibitors optimized for human therapy is needed.

*Emblica officinalis*, commonly known as Amla or the Indian gooseberry, is extensively used in the practice of Ayurveda, Indian traditional medicine, as a treatment for diabetes related complications [15]. Previous work has shown that crude aqueous extracts from Amla fruit delayed the onset and progression of cataracts and normalized diabetes-induced markers of lipid peroxidation and protein carbonyls [16], [17]. Moreover, these studies demonstrated that the active component(s) of the aqueous extract penetrate the lens and substantially delay the progression of cataracts through ALR2 inhibition. In this study we present the isolation and structure elucidation of the naturally occurring ALR2 inhibitor from *E. officinalis* fruit to be 1-*O*-galloyl-β-D-glucose (β-glucogallin). This well-known compound was first synthesized in 1918 by Emil Fisher and has since been shown to be a key bioprecursor found in numerous plants for larger, more complex tannins such as the gallotannins and ellagitannins [18]–[22]. We demonstrate that inhibition of substrate reduction is µM potent under saturating substrate conditions for both glucose and glyceraldehyde, and find that inhibition of substrate oxidation is noncompetitive for xylitol. Additionally, we show that β-glucogallin inhibition of ALR2 is specific over other aldo-keto reductases (AKRs) and active in an *ex vivo* transgenic lens organ culture, preventing the accumulation of sorbitol under hyperglycemic conditions.

## Results

### Isolation and Structure Elucidation of β-glucogallin

**Figure 1:**
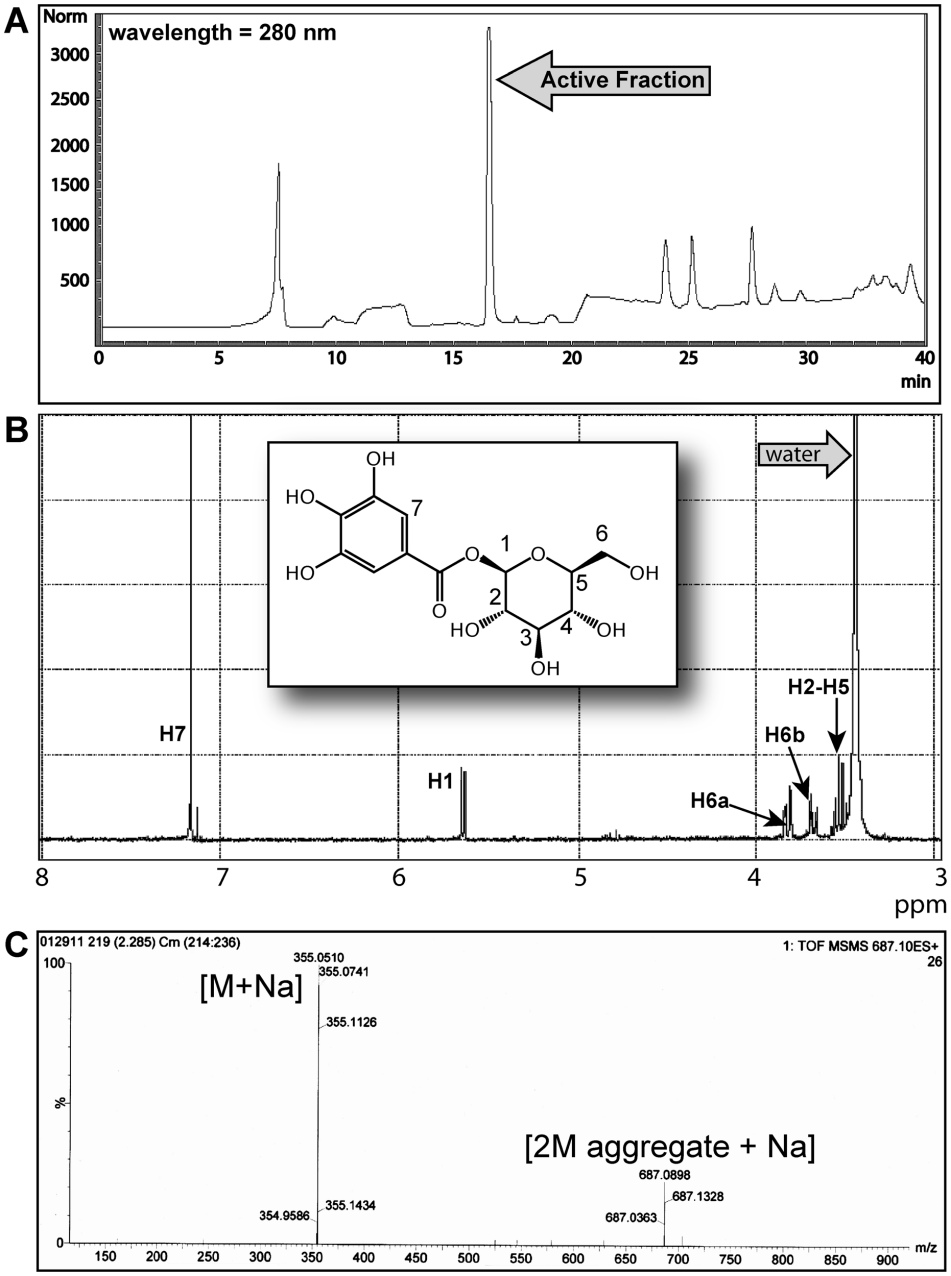
The elucidation of β-glucogallin as an active component and inhibitor of AKR1B1 from *E. officinalis.* (A) HPLC trace of pooled Sephadex® LH-20 fractions displaying activity against AKR1B1. The arrow indicates the fraction where activity against AKR1B1 appears to be localized. (B) The ^1^H NMR spectrum of the abundant active fraction from HPLC purification. The ^13^C spectrum and accurate mass data are shown in Figure S1 and S2 respectively. All of these data identify the active component as β-glucogallin. C) LC/MS/MS data indicate a peak at 687 *m/z* as a [2M aggregate + Na]. Therefore, the only compound present in this active fraction is β-glucogallin.

The aqueous extract of *E. officinalis* fruit has been shown to be rich in active constituents such as tannins and other antioxidants [16], [23], [24]. Thus, to facilitate identifying the active constituents against human AKR1B1, the lyophilized aqueous extract was further extracted with a 9∶1 (acetone:water) solution and subjected to Sephadex® LH-20 flash chromatography. Forty-five of the 150 column chromatography fractions showed significant activity against AKR1B1 *in vitro*. Material from the active fractions was further purified using bioassay-guided fractionation via C18 reversed phase HPLC, which revealed the highest inhibitory activity to be localized in one major fraction (Figure 1A). The ^1^H NMR spectrum of this fraction is shown in Figure 1B, (acetone-d_6_ and D_2_O): δ (ppm) 7.16 (2H, s), 5.65 (1H, d, J = 7.8), 3.85 (1H, dd, J = 12.4, 1.1), 3.70 (1H, dd, J = 12.4, 5.8), 3.41–3.48 (4H, m). The ^13^C NMR spectrum is shown in Figure S1, (DMSO-d_6_): δ (ppm) 165.0, 146.0, 139.3, 119.2, 109.4, 95.0, 78.3, 77.1, 73.1, 70.0, 61.0. In conjunction with NMR data, this fraction was subjected to LC-MS accurate mass analysis ([M+Na]^+^ experimental: 355.0624, calculated: 355.0606), which is consistent with the structure of β-glucogallin, agreeing with other structural reports for this compound in the literature (Figures 1, S1 and S2) [25], [26]. Additionally, the β-glucogallin active fraction showed an m/z peak at 687.089 (Figure 1C). Subsequent MS/MS analysis on ion m/z 687.089 confirmed this ion to be a β-glucogallin aggregate consisting of [2M aggregate + Na]^+^. Aggregation is a common phenomenon observed with saccharides during LC-MS analysis [27]. We observed no other stray peaks in any of the analytical spectra, confirming that the only compound present in the purified active fraction was β-glucogallin.

### Inhibition of Human Aldo-keto Reductases by β-glucogallin

**Figure 2:**
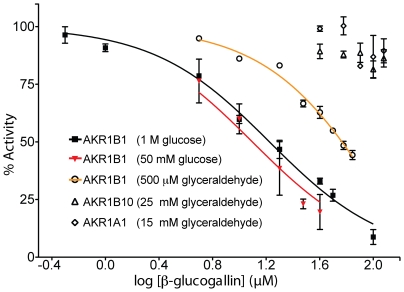
Inhibition of AKR substrate reduction by β-glucogallin. The IC_50_ was determined to be 58±3 µM (SEM) under saturating conditions of the shared substrate glyceraldehyde. β-glucogallin showed no activity against AKR1B10 and AKR1A1 at all concentrations tested under saturating conditions of glyceraldehyde as indicated. The IC_50_ was determined to be 17±1 µM under saturating conditions of the substrate glucose, and did not decrease significantly when the concentration of glucose was reduced 20-fold, IC_50_ = 13±1 µM). For all assays, inhibited enzyme activity was normalized to activity under the same conditions in the absence of inhibitor, assays were repeated in triplicate and error bars represent standard deviation from the mean. Where possible, GraphPad Prism software was used to fit normalized data to the enzyme inhibition model using nonlinear regression (method of least squares).

**Figure 3:**
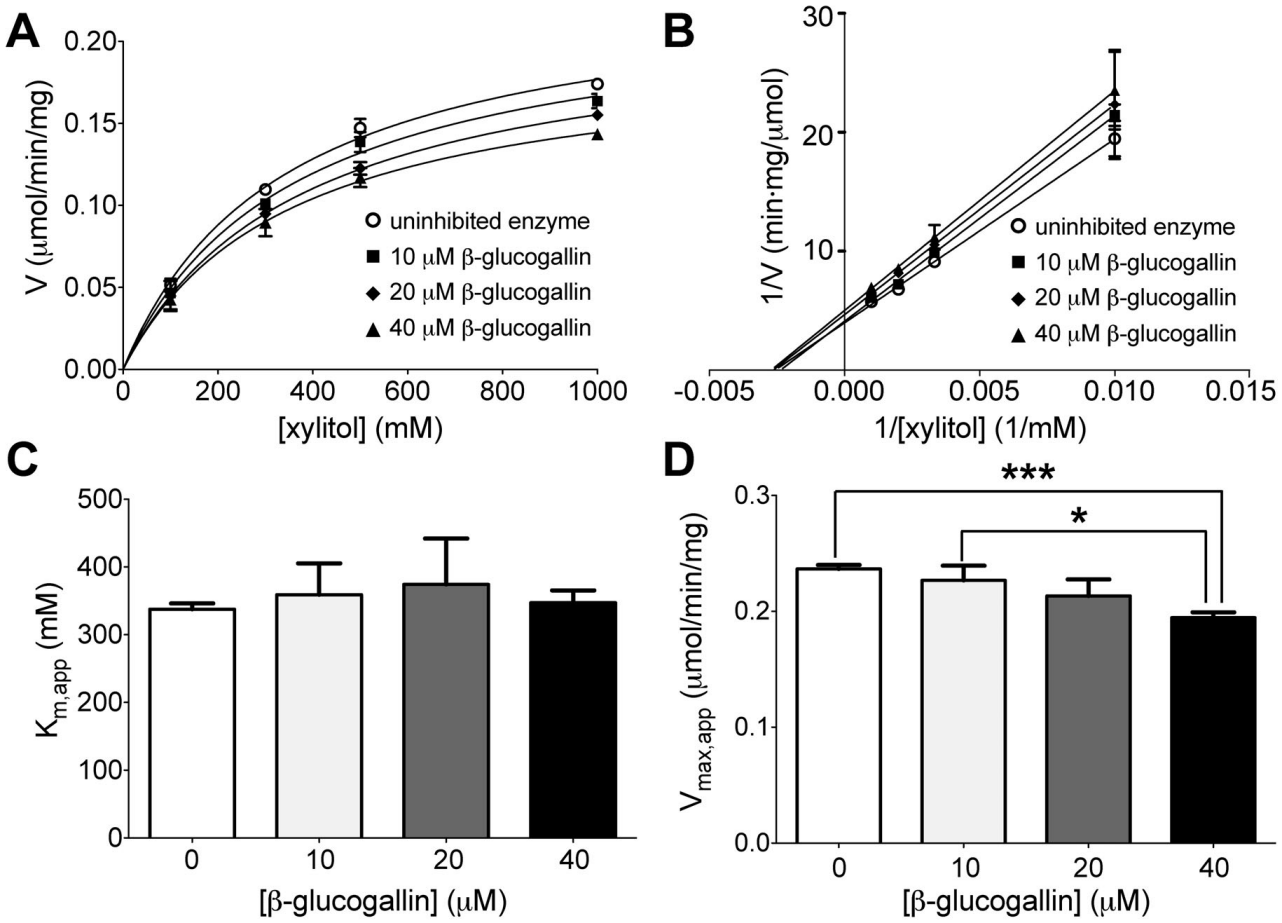
Inhibition of AKR1B1 substrate oxidation by β-glucogallin. A) Data were nonlinearly fit to the Michaelis-Menten model in the absence and presence of 0, 10, 20, and 40 µM β-glucogallin. B) Lineweaver-Burk plot of the same data, depicting increasing slope and y-intercept characteristic of noncompetitive inhibition. C) No significant changes were observed in the apparent K_m_ values. D) A decreasing trend was observed in the apparent V_max_ values, and the differences were found to be statistically significant as follows: *indicates *P* = 0.01, and ***indicates *P* = 0.0002. All assays were repeated in triplicate and error bars represent standard deviation from the mean. GraphPad Prism software was used to fit data to the Michaelis-Menten model using nonlinear regression (method of least squares) and to determine significance by column analysis of triplicates using the unpaired, two-tailed student’s t-test.

The inhibitory activity of purified β-glucogallin was determined for AKR1B1, as well as human aldose reductase-like protein 1 (AKR1B10) and human aldehyde reductase (AKR1A1) using the shared substrate glyceraldehyde to assess specificity amongst the structurally similar family of AKRs (Figure 2) [28], [29]. By nonlinear regression, the IC_50_ value of β-glucogallin for AKR1B1 was determined to be 58±3 µM (SEM). In contrast, negligible inhibition was observed when assays were conducted using AKR1B10 and AKR1A1. Using concentrations of glucose representing both enzyme-saturating (1 M) and physiological hyperglycemia (50 mM) conditions, the IC_50_ value was determined to be 17±1 and 13±1 µM (SEM), respectively (Figure 2). Detailed analysis of possible inhibition mechanisms using glucose as the variable substrate was not possible due to high and inconsistent rates of auto-oxidation in control reactions. Alternatively, assays were carried out in the “reverse” direction, using xylitol as the variable substrate with concomitant generation of NADPH. Under these conditions, Michaelis-Menten behavior was observed (Figure 3A) and inhibition appeared noncompetitive in the Lineweaver-Burk plot (Figure 3B) as the slopes of the linear fits are increased for the solutions with β-glucogallin present and the y-intercept increases with increasing concentrations. Further, by using nonlinear fits to the Michaelis-Menten model, no significant (*P*≥0.4) changes in the apparent K_m_ of AKR1B1 for xylitol were observed (Figure 3C) while the apparent V_max_ decreased significantly (*P*≤0.05) with increasing concentrations of β-glucogallin (Figure 3D), also in agreement with the model of noncompetitive inhibition. Therefore, using the noncompetitive inhibition model we found the inhibition constant (K_i_) for the oxidation of xylitol to be 170±20 µM (SEM).

Overall, these data confirm our initial studies, which showed the presence of an AKR1B1 inhibitor in the crude extracts from *E. officinalis* [16]. In addition, our results indicate a high degree of selectivity of β-glucogallin for AKR1B1 over the most abundant other members of the human AKR family.

### Computational Modeling of β-glucogallin with AKR1B1

**Figure 4:**
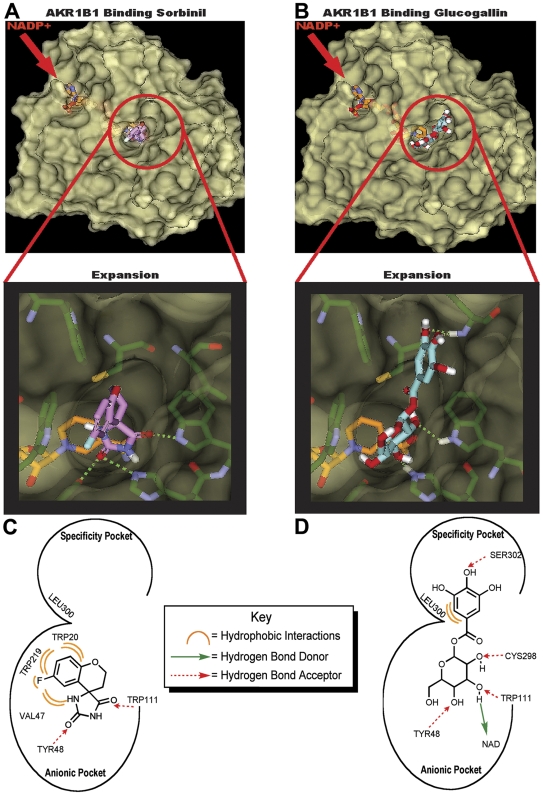
Computational modeling studies with β-glucogallin and AKR1B1. Modeling comparison between sorbinil (A) and β-glucogallin (B) showing ligands bound to AKR1B1. The solvent accessible surface depicts the active site anionic and specificity binding pockets, with expansion images to emphasize the detail of each pocket. The calculated binding energies for sorbinil and β-glucogallin are –32 kcal/mol and –44 kcal/mol, respectively. The active site interactions are summarized for sorbinil (C) and β-glucogallin (D) and were identified using Discovery Studio software v2.5.5 (Accelrys) and Ligand Scout v2.3. Based on the calculated binding energies and number of active site interactions β-glucogallin appears to bind AKR1B1 as effectively as the known active inhibitor sorbinil.

The active site of AKR1B1 consists of two major pockets, the so-called “anionic” pocket and the “specificity” pocket [30]. The active site of AKR1B1 can accommodate a multitude of substrates due to the flexibility of the “specificity pocket” residues [31]. Interestingly, deposited XRAY crystallography structures of bound AKR inhibitors demonstrate that competitive, uncompetitive, and noncompetitive inhibitors alike bind in the active site [32]–[34]. To investigate how β-glucogallin may bind AKR1B1, we carried out computational molecular docking studies using Discovery Studio software and Ligand Scout. Using Discovery Studio, a search of the AKR1B1 crystal structure surface was conducted to identify available binding sites that would accommodate β-glucogallin using flexible and rigid docking. The AKR1B1 active site was identified as the most likely binding site due to the available area, high-scoring complexes, DOCK score, binding energy, and the visualized protein ligand binding conformation. Further, β-glucogallin bound the active site of AKR1B1 obtaining a more favorable predicted binding energy than sorbinil (–44 kcal/mol and –32 kcal/mol, respectively), a known active site inhibitor of AKR1B1 (Figure 4) [30], [35]. Sorbinil only binds to the anionic pocket and has been shown to inhibit ALR1 and ALR2 equally. However, other inhibitors that bind to both the “anionic” and “specificity” pockets show specificity for ALR2 [31], [33]. β-glucogallin occupies both the “anionic” and “specificity” pockets resulting in more active site interactions (Figure 4C–D). Notably, the glucose moiety can form key hydrogen bonds with active site residues in the anionic pocket, including: Tyr48, Trp111, His110, Cys298, and the bound natural cofactor NADP+ (Figure 4D). Additionally, the phenolic moiety of β-glucogallin extends into the “specificity” pocket, potentially forming hydrogen bonds with Ser302 and obtaining favorable hydrophobic interactions with Leu300. These data suggest that β-glucogallin can bind AKR1B1 as effectively as sorbinil, potentially with greater affinity and specificity.

### Lens Organ Culture Study with β-glucogallin

**Figure 5:**
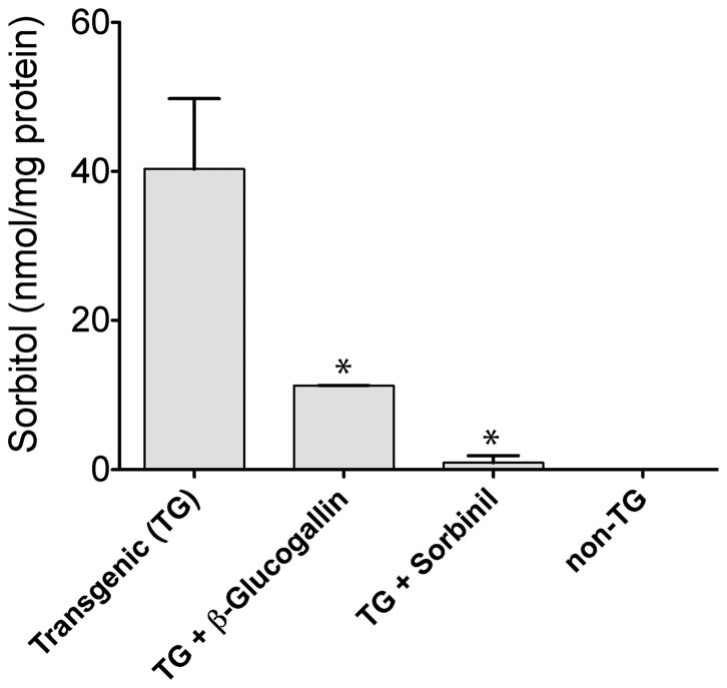
β-glucogallin prevents sorbitol accumulation in transgenic (TG) human AKR1B1 expressing lenses *ex-vivo*. Lenses extracted from TG-animals were cultured under hyperglycemic conditions in the presence or absence β-glucogallin (30 µM) or sorbinil (10 µM) over 72 hours. β-glucogallin showed potent activity preventing sorbitol accumulation by 73% as compared to untreated lenses. Comparably, the positive control sorbinil inhibited sorbitol accumulation by 97%. The amount of sorbitol in the non-transgenic controls was below the limit of detection. GraphPad Prism software was used to compare sorbitol accumulation in treated lenses to untreated controls by column analysis of duplicates using the unpaired, one-tailed student’s t-test. The * symbol denotes a statistically significant difference (*P*<0.05) from untreated TG lenses.

To confirm our *in vitro* data, we utilized a transgenic mouse model, which overexpresses AKR1B1 specifically in the lens. Lenses were dissected from both transgenic and non-transgenic mice and cultured *ex-vivo* under high glucose conditions resulting in sorbitol accumulation in transgenic lenses over 72 hours (Figure 5). Transgenic lenses treated with 30 µM β-glucogallin under the same conditions significantly (*P≤0.05) inhibited sorbitol accumulation by 73% (Figure 5). Similarly, lenses treated with the positive control sorbinil (10 µM) inhibited sorbitol accumulation by 97% (*P≤0.05). No sorbitol accumulation was observed in the non-transgenic mouse control lenses. These data not only indicate that β-glucogallin can penetrate lens tissues *ex-vivo,* but can successfully inhibit AKR1B1 under hyperglycemic conditions, preventing the accumulation of sorbitol and subsequent osmotic stress.

## Discussion

Due to its involvement in the development of diabetic complications, AKR1B1 is an attractive drug target in the clinical management of secondary complications of diabetes including cataract and retinopathy. As a result, AKR1B1 has been extensively investigated using structure based drug design to develop potent inhibitors [35]. Thus far, all of these man-made inhibitors have failed to realize clinically useful therapies due to toxicity and/or poor tissue penetration [36], [37]. The Amla fruit has been used in Ayurvedic medicinal preparations for thousands of years as a safe and effective long-term treatment of diabetic complications and other diseases of the eye [15]. We hypothesized that this plant would provide inhibitors of AKR1B1 that could lead to the identification of lead compounds that could be exploited for novel drug development. In support of our hypothesis we previously demonstrated that the crude aqueous extract produced from fruits of *E. officinalis* suppressed the onset and development of cataract in a rat model [16], [17]. Furthermore, we demonstrated that this extract could inhibit AKR1B1 enzyme activity, suggesting that the efficacious effects against cataract formation stem from inhibition of AKR1B1, preventing sorbitol accumulation and subsequent osmotic stress.

In this study we successfully isolated an abundant fraction from *E. officinalis* using bioassay-guided isolation. This major fraction proved to be active against AKR1B1, which was not observed with other fractions. Therefore, the inhibitory activity appears to be localized as shown in Figure 1A. Structural analysis of the active fraction revealed the active component to be β-glucogallin (Figures 1, S1, and S2). Interestingly, β-glucogallin is a well-known plant metabolite and major biosynthetic precursor for the production of larger more complex hydrolysable tannoids [18]–[22]. Nevertheless, β-glucogallin has not been previously reported to have significant biological activity against human disease. Indeed, we show β-glucogallin is a relatively potent and selective inhibitor of AKR1B1. Using the substrate glyceraldehyde, β-glucogallin inhibited AKR1B1 with an IC_50_ value of 58±3 µM (SEM) (Figure 2). Structurally, AKR1B1 is similar to several AKRs in human tissues, including the aldose reductase-like protein 1 (AKR1B10) and glucuronate reductase (AKR1A1; also known as aldehyde reductase). Still, only negligible and non-dose-responsive inhibition of AKR1B10 and AKR1A1 activity was observed even with excessive concentrations of β-glucogallin (≥ 100 µM). Importantly, among these human AKRs, AKR1B1 is unique in its ability to catalyze the NADPH-dependent conversion of glucose to sorbitol [38]. While the galloyl moiety, which occupies the “specificity” pocket may confer specificity for ALR2 over ALR1 [31]; we theorize that the glucose moiety of β-glucogallin may be the factor that confers specificity for AKR1B1 observed among the ALR2 family. This is desirable, as non-specific inhibition can result in toxicity and other adverse side effects.

To shed light on the pharmacological relevance of AKR1B1 inhibition by β-glucogallin, assays were conducted using conditions that mimic either saturating or physiological (non-saturating) concentrations of glucose. Since the observed potency of β-glucogallin did not appreciably shift across a 20-fold range in substrate concentration, *i.e*. 17±2 µM in 1 M glucose *vs*. 13±3 µM in 50 mM glucose (95% CI), the pattern of inhibition for the reduction of glucose observed was not competitive (Figure 2). Accordingly, β-glucogallin is similar to other ALR2 active site inhibitors, which show uncompetitive to noncompetitive inhibition patterns when measured in the direction of aldehyde reduction [32]. Inhibitor binding to ALR2 can give rise to complex kinetic parameters depending on the range of enzyme states recognized by the inhibitor and their associated binding affinities, *e.g*., E, E-NADPH, or E-NADP^+^ and the conformational isomers associated with each of these [23]. Future studies will be needed to establish these binding parameters.

Our computational molecular docking studies indicate that β-glucogallin binds favorably to the active site of AKR1B1 (Figure 4). The sugar moiety occupies the anionic site and appears to mimic natural substrates of AKR1B1 by forming hydrogen bonds with key residues that are necessary for catalytic function, as well as the bound cofactor NADPH. The phenolic moiety extends into the specificity pocket to form hydrophobic interactions as well as hydrogen bonds with residues Leu300 and Ser302, respectively. Therefore, we propose that β-glucogallin would inhibit free glucose binding at the active site of AKR1B1, preventing sorbitol production under hyperglycemic conditions such as in patients suffering from diabetic eye disease. This is substantiated by our observation that 30 µM β-glucogallin repressed sorbitol accumulation by 73% in AKR1B1 transgenic mouse lenses incubated under high glucose (27.5 mM) conditions that mimic hyperglycemia in humans (Figure 5).

In conclusion, natural products such as β-glucogallin are attractive as therapeutic leads in the treatment of diabetic complications, since their low toxicity allows them to be used as long-term prophylactics. In this context, we have been investigating the promise of dietary sources of aldose reductase inhibitors to prevent diabetic cataract in animals. Thus far, we have successfully identified and purified the most active AKR1B1 inhibitor from *E. officinalis* as β-glucogallin, and have demonstrated its inhibitory efficacy *in vitro* and in lens tissues in an *ex-vivo* model. Finally, this study supports the continued development of β-glucogallin as a therapeutic treatment against diabetic cataracts.

## Materials and Methods

### General Methods

Semi-preparative and analytical HPLC were performed using an Agilent 1100 HPLC quaternary pump fitted with the appropriate column as described below. HPLC solvents were filtered through a 0.45 µm filter and degassed before use. HPLC-grade solvents, all other chemical solvents, and biological reagents described were purchased from Fisher Scientific (Pittsburgh, PA). Sorbinil was a kind gift from Pfizer (New York, NY). NMR data were collected using a Bruker Avance III 400 (^1^H 400 MHz, ^13^C 100 MHz). LC-MS analyses were conducted using a Micromass Q-TOF-2 Micromass spectrometer equipped with lock spray and an Agilent 1100 HPLC capillary pump fitted with a C_18_ Mass Spec column from Vydac (1X150mm). The MS was run in positive mode with a capillary voltage of 2.5 kV and a cone voltage of 30 V. Accurate mass measurements were performed using leucine enkephalin as a lock mass and data were processed using MassLynx 4.1. Spectroscopic kinetic analyses were performed using a Cary Bio 100 UV/Vis spectrophotometer (Agilent) in quartz cuvettes in conjunction Cary WinUV software. Computational molecular docking was performed using Discovery Studio software v2.5.5 (Accelrys) and Ligand Scout v2.3 (MacResearch).

### Animal Protocol

The authors confirm that use of mice in these studies adheres to policies in the Guide for the Care and Use of Laboratory Animals, the United States Public Health Service Policy on the Humane Care and Use of Laboratory Animals, and the U.S. Animal Welfare Act. Approval for the use of mice was granted by the University of Colorado-AMC Capitol Institutional Animal Care and use Committee (IACUC Protocol Number: B-85109(04)1C).

### Purification of AKR1B1 Inhibitors from *E. officinalis*

Fresh fruits of *E. officinalis* were collected from the local market in Hyderabad (India) and the pericarp of the fruit was lyophilized. An aqueous extract was prepared from the powdered material as previously described and lyophilized [16]. The lyophilized powder (10 g) was further extracted with 200 mL of a 9∶1 (acetone: water) solution and filtered. The aqueous acetone filtrate was loaded onto the column (54 cm×4 cm) packed with 100 g of Sephadex® LH-20 (Sigma-Aldrich; St. Louis, MO) previously saturated with absolute ethanol (500 mL) over 24 hr. The crude aqueous acetone extract was eluted sequentially with 600 mL absolute ethanol, 500 mL absolute ethanol and methanol (1∶1), and 1,200 mL of methanol, using a flow rate of 1.2 mL/min. The resulting eluent was collected into 15 mL fractions, which were evaporated under a stream of nitrogen and stored as a dry residue at 4°C. Fractions determined to be active against AKR1B1 were purified by HPLC using a Phenomenex Luna C18 reversed phase semi-prep column (5 µm, 100 Å, 10×250 mm) fitted with a guard column. 180 µg per injection was loaded onto the column and elution was performed with a flow rate of 1.5 mL/min using a linear gradient of 5–30% acetonitrile: water and a run time of 35 min, monitored by absorbance at 280 nm. Fractions corresponding to absorbance peaks were pooled and evaporated to dryness under a stream of nitrogen.

### Structure Elucidation

The active pure fraction was subjected to ^1^H and ^13^C NMR as well as LC-MS for structural determination. The ^1^H NMR (400 MHz) spectrum was obtained in acetone-d_6_ using a drop of D_2_O to aid in dissolution. The ^13^C NMR (100 MHz) spectrum was obtained in DMSO-d_6_. LC-MS analysis was conducted using an initial isocratic mobile phase of 5% acetonitrile: water held for 2 minutes, followed by a linear gradient to 60% acetonitrile: water over 8 minutes. The flow rate was maintained at 50 µL/min. Following structural elucidation, solutions of pure β-glucogallin in water were prepared for *in-vitro* and *ex-vivo* analysis.

### Purification of Recombinant Human Aldo Keto Reductases

Recombinant human aldose reductase (AKR1B1) was over-expressed in *E. coli* and purified from bacterial cultures as previously described [16], [39]. By a similar method, quantities of AKR1B10 (human aldose reductase-like protein 1) and AKR1A1 (human aldehyde reductase) were purified from *E. coli* expression cultures. For all recombinant AKR enzymes used in this study, the enzymes were purified to an apparently homogeneous state as judged by the appearance of a single protein band following SDS-PAGE of the purified material.

### Inhibition of AKR1B1 and Other Members of the AKR Family

Crude plant fractions were dissolved in DMSO at 20 µg/ml and final concentration of DMSO was kept below 1% in enzyme assay mixtures. Purified β-glucogallin was dissolved in water and stored at –20°C until use. The activity of AKR1B1, AKR1B10 and AKR1A1 with glyceraldehyde and the activity of AKR1B1 with glucose was determined by the method of Hayman and Kinoshita [40]. Briefly, the K_m_ for glyceraldehyde was determined by combining purified enzyme with a solution containing saturating (175 µM) NADPH and varying concentrations of glyceraldehyde in KAB buffer (50 mM Hepes (pH 7.5), 150 mM NaCl, 1 mM DTT, 10 mM MgCl_2_). Solutions were kept on ice before mixing, then incubated at 37°C and initial velocities were monitored by following the absorbance extinction at 340 nm, corresponding to NADPH oxidation (stoichiometrically, one mole of NADPH is oxidized to convert one mole of substrate). For reactions with glucose, a final concentration of 375 µM NADPH was used to provide saturating conditions for the larger concentration of enzyme required to afford measurable reaction rates.

IC_50_ assays were conducted similarly using a saturating concentration of substrate, defined to be at least ten times greater than the observed K_m_ for that enzyme-substrate pair, as well as physiological hyperglycemic conditions (50 mM glucose). For each concentration of β-glucogallin used, enzyme activity was normalized to that of activity using the same conditions in the absence of inhibitor. The activity of AKR1B1 in the direction of substrate oxidation was determined with xylitol [41]. Briefly, assays were conducted as above using a final concentration of 2.5 mM NADP^+^ and varying concentrations of xylitol in 50 mM phosphate buffer, pH 7.0. In this case, enzyme activity was monitored on the same principle, using the absorbance increase at 340 nm corresponding to NADP^+^ reduction.

Enzyme-free solutions were used to determine the mean and standard deviation of the background signal for oxidation of NADPH and reduction of NADP^+^ and means were subtracted from observed signals as appropriate. All kinetic experiments were repeated in triplicate and GraphPad Prism software was used to fit normalized data to the enzyme inhibition model for IC_50_ values, the Michaelis-Menten model for K_m_ and V_max_ values, and the noncompetitive inhibition model for the K_i_ value using nonlinear regression (method of least squares). Double-reciprocal (Lineweaver-Burk) data was linearly fit by the method of least squares in order to compare slope and intercept values, however, these plots were not utilized quantitatively to determine Michaelis-Menten constants.

### *Ex-vivo* Lens Organ Culture Studies

Transgenic mice designed for lens-specific expression of AKR1B1 were produced by standard methods on a C57BL6 strain background. The current study was carried out using the strain designated PAR40, which expresses AKR1B1 at approximately 30 milliunits/mg protein when cleared lens homogenates were assayed under standard conditions [42]; in contrast, AKR1B1 activity in lens homogenates from nontransgenic control was practically undetectable. Lenses for organ culture studies, which were harvested from 8–12 week old transgenic and nontransgenic control mice, were morphologically normal. Mice were euthanized under CO_2_, eyes were removed and lenses dissected into Dulbecco’s Modified Eagle’s Medium (DMEM, D5030, Sigma) supplemented with insulin/transferring/selenium (Sigma), penicillin-streptomycin and fungizone (Gibco) and were incubated in the presence of 95% O_2_, 5% CO_2_ at 37°C. Lenses that remained clear after 24 h incubation were then switched to either DMEM containing 5.5 mM glucose (basal glucose) or DMEM containing 27.5 mM glucose (high glucose) in the presence or absence of β-glucogallin. Presence of lens opacities was monitored daily. At the end of the experiment, sorbitol content in the lenses was determined. Sorbitol content in cultured lenses was estimated by an enzymatic method [43]. The total soluble protein content was estimated by BCA method using BSA as the standard.

### Computational Modeling of β-glucogallin with AKR1B1

To investigate how β-glucogallin is able to bind AKR1B1, we carried out computational-based molecular docking studies. The CHARMM force field was applied to the 0.93 Å AKR1B1 (PDB: 1PWM) crystal structure [44], water molecules were removed, and residues were corrected for physiological pH. The binding site was defined as whole residues within an 8 Å radius subset encompassing the active site. LigandFit [45] was used for the molecular docking of β-glucogallin and sorbinil into the defined binding site of AKR1B1. Grid resolution was set to 0.5 Å and electrostatic energy was included in the calculation of the ligand internal energy. In order to avoid identical conformations, a root mean square deviation threshold of 1.5 Å and a score threshold of 20 kcal/mol were used. Fifty structural outputs were specified and the identification of a docked conformation was followed by a minimization using the conjugate gradient method to a convergence of 0.001 kcal/mol to optimize ligand-protein interactions. Active site interactions were determined utilizing the Structure Monitor in Discovery Studio in conjunction with the Create Pharmacophore protocol in LigandScout. The top-ranked conformations for each docked complex were selected on the basis of the following criteria: the calculated binding energy and the potential interactions between the protein and ligand.

## Supporting Information

Figure S1 **The ^13^C NMR spectrum of β-glucogallin (DMSO-d_6_): δ (ppm) 165.0, 146.0, 139.3, 119.2, 109.4, 95.0, 78.3, 77.1, 73.1, 70.0, 61.0.**
(TIFF)

Figure S2 **The LCMS accurate mass analysis of β-glucogallin.**
(TIF)
