# Supplementary figures and images for: The Isolation and Characterization of β-Glucogallin as a Novel Aldose Reductase Inhibitor from Emblica officinalis
Source: PLoS One. 2012 Apr 2;7(4):e31399. doi: 10.1371/journal.pone.0031399 (PMC3317655; doi:10.1371/journal.pone.0031399)

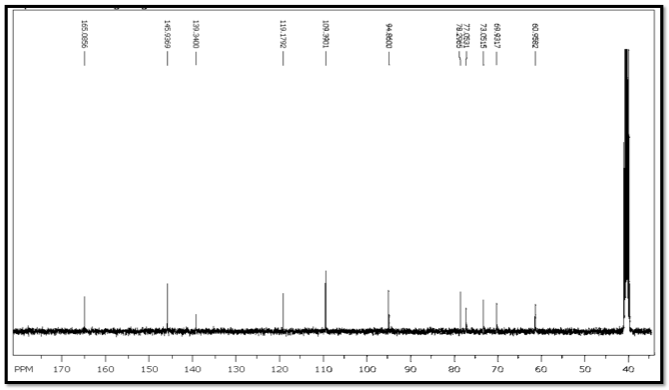

Supplement: Figure S1 — The 13C NMR spectrum of β-glucogallin (DMSO-d6): δ (ppm) 165.0, 146.0, 139.3, 119.2, 109.4, 95.0, 78.3, 77.1, 73.1, 70.0, 61.0. (TIFF) [file pone.0031399.s001.tif]

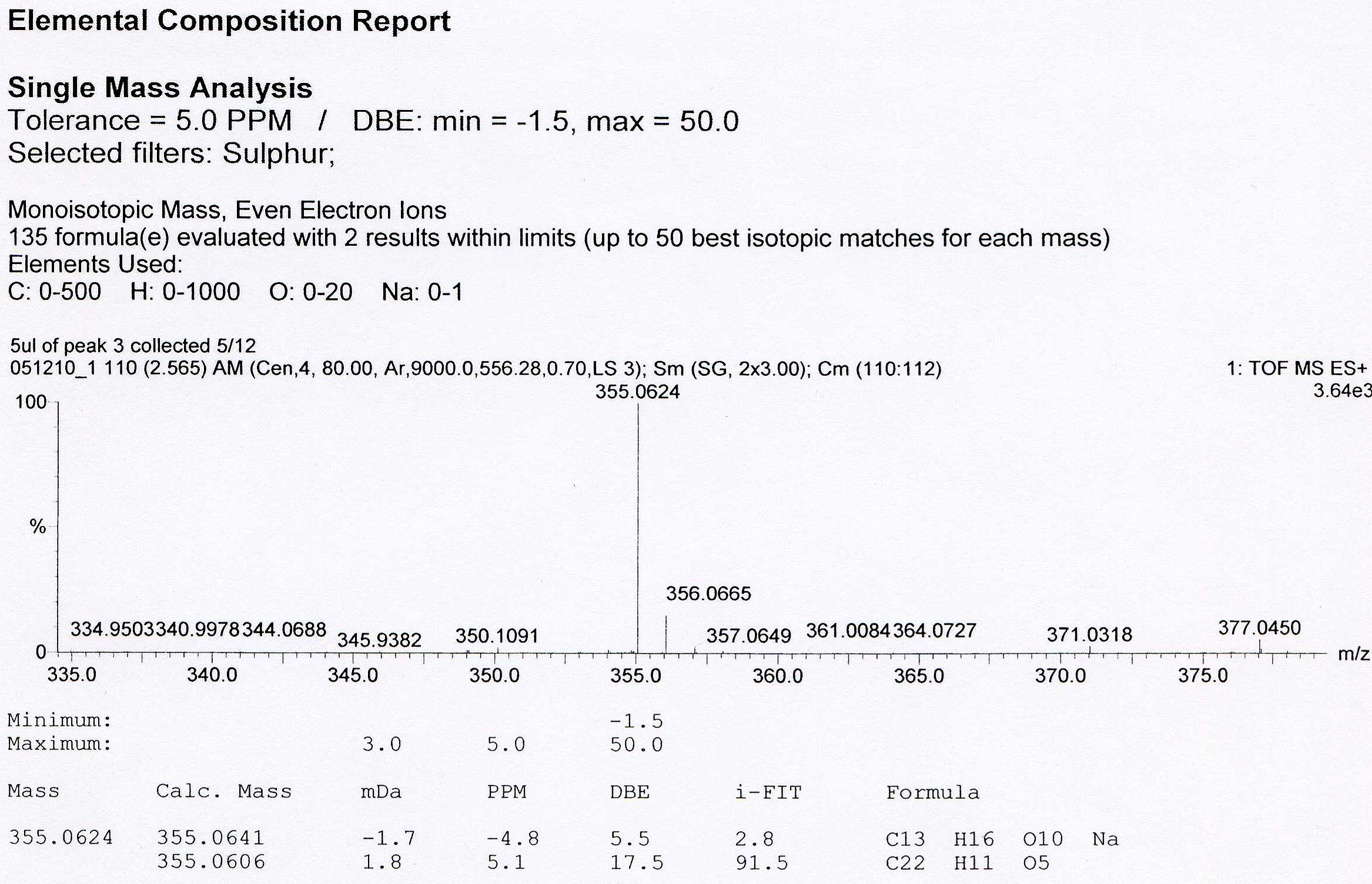

Supplement: Figure S2 — The LCMS accurate mass analysis of β-glucogallin. (TIF) [file pone.0031399.s002.tif]
